# Potentially inappropriate primary care prescribing in people with chronic kidney disease: a cross-sectional analysis of a large population cohort

**DOI:** 10.3399/BJGP.2020.0871

**Published:** 2021-05-05

**Authors:** Clare MacRae, Stewart Mercer, Bruce Guthrie

**Affiliations:** Usher Institute of Population Health Sciences and Informatics, University of Edinburgh, Edinburgh.; Usher Institute of Population Health Sciences and Informatics, University of Edinburgh, Edinburgh.; Usher Institute of Population Health Sciences and Informatics, University of Edinburgh, Edinburgh.

**Keywords:** chronic kidney diseases, epidemiology, general practice, potentially inappropriate prescribing, renal impairment

## Abstract

**Background:**

Many drugs should be avoided or require dose-adjustment in chronic kidney disease (CKD). Previous estimates of potentially inappropriate prescribing rates have been based on data on a limited number of drugs, and mainly in secondary care settings.

**Aim:**

To determine the prevalence of contraindicated and potentially inappropriate primary care prescribing in a complete population of people with known CKD.

**Design and setting:**

Cross-sectional study of prescribing patterns in a complete geographical population of people with CKD, defined using laboratory data.

**Method:**

Drugs were organised by British National Formulary advice — contraindicated drugs: ‘avoid’; potentially high-risk (PHR) drugs: ‘avoid if possible’; and dose-inappropriate (DI) drugs: ‘dose exceeded recommended maximums’. CKD was defined as estimated glomerular filtration rate (eGFR) *≤*60 ml/min/1.73 m^2^ for *>*3 months.

**Results:**

In total, 28 489 people with CKD were included in the analysis, of whom 70.1% had CKD stage 3a, 22.4% CKD stage 3b, 5.9% CKD stage 4, and 1.5% CKD stage 5. A total of 3.9% (95% confidence interval [CI] = 3.7 to 4.1) of people with CKD stages 3a–5 were prescribed *≥*1 contraindicated drug, 24.3% (95% CI = 23.8 to 24.8) *≥*1 PHR drug, and 15.2% (95% CI = 14.8 to 15.6) *≥*1 DI drug. Contraindicated drugs differed in prevalence by CKD stage and were most commonly prescribed in CKD stage 4, with a prevalence of 36.0% (95% CI = 33.7 to 38.2). PHR drugs were commonly prescribed in all CKD stages, ranging from 19.4% (95% CI = 17.6 to 21.3) in CKD stage 4 to 25.1% (95% CI = 24.5 to 25.7) in CKD stage 3a. DI drugs were most commonly prescribed in CKD stage 4 (26.4%, 95% CI = 24.3 to 28.6).

**Conclusion:**

Potentially inappropriate prescribing is common at all stages of CKD. Development and evaluation of interventions to improve prescribing safety in this high-risk population are needed.

## INTRODUCTION

Chronic kidney disease (CKD) is an abnormality in kidney structure or function, present for *>*3 months, defined by cause, glomerular filtration rate (GFR), and albuminuria category.^1^^,^^2^ The Global Burden of Disease study estimates worldwide prevalence of all-stage age-standardised CKD at 9.1%,^3^ making it a significant public health problem. CKD encompasses a heterogeneous group of disorders^4^ of varying severity and rate of progression.^5^ Although the proportion of individuals with CKD who develop end-stage renal dysfunction (ESRD) and require renal replacement therapy (RRT) or transplantation is small,^6^ CKD is an important risk factor for cardiovascular disease (CVD) and all-cause mortality,^6^^,^^7^ and significantly drives healthcare costs.^1^^,^^7^^–^^9^ Good clinical care, including the adjustment of medications according to renal function, and avoiding medications that increase the risk of adverse outcomes, can slow progression and reduce morbidity.^1^^,^^10^ GPs are at the front line in early identification and management of CKD.^9^ In the UK, for example, almost all long-term prescribing and medication reviews occur in the primary care setting,^11^^,^^12^ making primary care a key target for interventions to improve prescribing safety in CKD.

CKD prevalence rises sharply with increasing age, and comorbidity and polypharmacy are therefore common in people with CKD.^4^^,^^13^ Clinical management is often complicated by multiple physicians being simultaneously involved in patient care.^14^ Adverse drug reactions (ADRs) are unintended harmful events attributed to the use of medicines.^15^ Individuals with CKD are at particularly high risk of ADRs,^16^ owing to altered pharmacokinetics and pharmacodynamics that predispose to drug accumulation, as well as direct nephrotoxicity.^4^^,^^17^ People with CKD are at increased risk of drug-related acute kidney injury (AKI), and have the poorest AKI outcomes in terms of morbidity, mortality, and additional loss of kidney function, with accelerated progression to ESRD.^17^

Most studies to date have focused on potentially inappropriate prescribing in all adults with CKD in secondary care, with fewer studies examining community prescribing of a wide range of drugs.^18^^–^^21^ The aim of the current study was to examine the prevalence of potentially inappropriate prescribing in a population cohort of people with CKD.

## METHOD

The study design was a retrospective population-based analysis of all residents of two Scottish health boards aged *≥*18 years with laboratory-confirmed CKD. Health care in Scotland is provided free at the point of use by the NHS. Registration with a single general practice is required. This provides primary medical care, acts as a gatekeeper to secondary care, and prescribes virtually all community-dispensed medicines, including those recommended by specialists (who only prescribe highly specialist drugs such as cancer chemotherapy and some biologics). Linkage between datasets was performed at a patient level using the community health index (CHI) number, the NHS Scotland unique patient identifier. Linked data used in analysis included demography, laboratory data to define CKD, and community-dispensed prescriptions. Every dispensed prescription was provided with 100% allocation of prescriptions to individuals. Data were provided by the University of Dundee Health Informatics Centre (HIC) (https://www.dundee.ac.uk/hic). All data analysis was performed using anonymised data held in the ISO270001 and NHS Scotland-accredited HIC Safe Haven.

**Table:** How this fits in

GPs are at the front line in identification and management of chronic kidney disease (CKD), and in the UK almost all long-term prescribing and medication reviews occur in the primary care setting, making this a key target for interventions to improve prescribing safety in CKD. Several studies refer to potentially inappropriate prescribing in secondary care, but little is known about the prevalence of potentially inappropriate prescribing in CKD for a wide range of drugs in primary care. This study finds that potentially inappropriate prescribing in primary care is common at all stages of CKD, and existing recommendations for prescribing in renal impairment are often non-specific and relatively unhelpful to clinicians. There is a need to improve understanding of the benefit–harm balance of prescribing in renal impairment, and to develop interventions to improve prescribing safety.

CKD status and stage were determined using laboratory-calculated estimated glomerular filtration rate (eGFR) values, calculated by the hospital laboratory carrying out the creatinine measurement using isotope dilution mass spectrometry standardised creatinine values, traceable to National Institute of Standards and Technology Standard Reference Materials 914, using the abbreviated Modification of Diet in Renal Disease (MDRD) equation.^22^ A cross-sectional cohort of permanently registered residents with CKD was defined on 31 March 2018, using the most recent eGFR values. CKD was defined as most recent eGFR *<*60 ml/min/1.73 m^2^, a previous eGFR *<*60 ml/min/1.73 m^2^ *>*84 days previously, and no intervening eGFR values *≥*60 ml/min/1.73 m^2^. CKD stage was defined as CKD stage 3a (mild) for eGFR 45–59 ml/min/1.73 m^2^, CKD stage 3b (moderate) for eGFR 30–44 ml/min/1.73 m^2^, CKD stage 4 (severe) for eGFR 15–29 ml/min/1.73 m^2^, and CKD stage 5 (ESRD) for eGFR *<*15 ml/min/1.73 m^2^. Categorisation into mild, moderate, severe, and end-stage groupings was done to allow application of British National Formulary (BNF) prescribing standards, because the majority of BNF ‘renal impairment’ warnings referred to these terms rather than eGFR.

**Figure 1.**
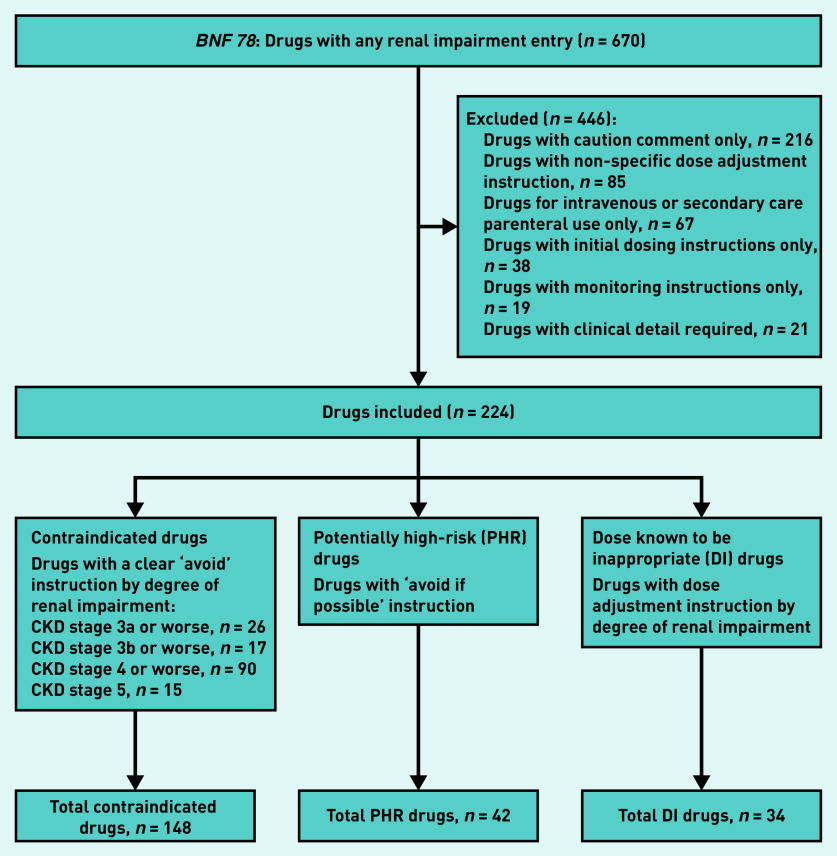
*Drug inclusion chart.* *BNF = British National Formulary. CKD = chronic kidney disease.*

All drugs with a renal impairment warning in *BNF 78* September 2019 to March 2020^23^ were identified, and warnings were categorised into three groups (Figure 1). Contraindicated drugs were those where the warning explicitly stated to avoid the drug at particular levels of renal function. Potentially high-risk (PHR) drugs were those where the warning stated ‘avoid if possible’ in all stages of CKD. Dose known to be inappropriate (DI) drugs were identified where prescribed drug strength exceeded the maximum recommended dose in the BNF for a given level of renal function. A drug in any three of these groups was considered to be currently prescribed if dispensed in the 84 days before the cohort index date of 31 March 2018.

The authors analysed the prevalence of current prescription of all included drugs in people with CKD stage 3a or worse within the total population calculated according to National Records of Scotland 2018 mid-year population estimates,^24^ and stratified by CKD status, and 95% confidence intervals (CIs) were calculated. Statistical analyses were undertaken using IBM SPSS Statistics (version 22), and plots were created in GraphPad Prism (version 9.2).

## RESULTS

In total, there were 28 489 individuals aged *≥*18 years with known CKD, based on the most recent laboratory evaluation and registered with a GP in the region on 31 March 2018 (Table 1), representing 4.4% of the total population of 644 080 people.^24^ Between 1 January 2006 and 31 March 2018, 488 268 adults aged *≥*18 years had *≥*1 eGFR value reported. Of those, 27 931 had only one eGFR and so could not be evaluated, leaving 460 337 who had *≥*2 eGFR values and could be evaluated for CKD. Of this group, 84.0% of those aged 65–74 years, and 90.0% of those aged *≥*75 years were evaluable (data not shown). In all, 19 977 (70.1% of all people with CKD) had CKD stage 3a, 6383 (22.4%) had CKD stage 3b, 1693 (5.9%) had CKD stage 4, and 436 (1.5%) had CKD stage 5 (Table 1). Mean age was similar throughout CKD cohorts, ranging from 72.3 years (standard deviation [SD] 14.4) in CKD stage 5 to 79.4 years (SD 10.9) in CKD stage 3b. Female sex was more common in all CKD stages except CKD stage 5. People with CKD across all stages were most commonly in the second and third quintile for Scottish Index of Multiple Deprivation (SIMD) (1 being the least and 5 being the most deprived).

**Table 1.** Study and population characteristics

	**Any CKD stage, *n*= 28 489 (4.4% of 644 080)^24^**	**CKD stage 3a (eGFR 45–59), *n*= 19 977**	**CKD stage 3b (eGFR 30–44), *n*= 6383**	**CKD stage 4 (eGFR 15–29), *n*= 1693**	**CKD stage 5 (eGFR <15), *n*= 436**
**Age, years, mean (SD)^a^**	74.8 (12.3)	73.1 (12.2)	79.4 (10.9)	78.2 (13.0)	72.3 (14.4)
18–24	21 (0.1)	14 (0.1)	5 (0.1)	2 (0.1)	0 (0.0)
25–34	152 (0.5)	115 (0.6)	18 (0.3)	10 (0.6)	9 (2.1)
35–44	369 (1.3)	284 (1.4)	38 (0.6)	33 (1.9)	14 (3.2)
45–54	1367 (4.8)	1157 (5.8)	125 (2.0)	51 (3.0)	34 (7.8)
55–64	3285 (11.5)	2755 (13.8)	348 (5.5)	126 (7.4)	56 (12.8)
65–74	7509 (26.4)	5859 (29.3)	1240 (19.4)	308 (18.2)	102 (23.4)
75–84	9478 (33.3)	6386 (32.0)	2399 (37.6)	558 (33.0)	135 (31.0)
≥85	6308 (22.1)	3407 (17.1)	2210 (34.6)	605 (35.7)	86 (19.7)

**Sex**					
Female	17 768 (62.4)	12 487 (62.5)	4085 (64.0)	985 (58.2)	211 (48.4)

**Socioeconomic status by SIMD quintile^b^**					
1 (least deprived)	4981 (17.5)	3456 (17.3)	1125 (17.6)	313 (18.5)	87 (20.0)
2	6288 (22.1)	4312 (21.6)	1442 (22.6)	418 (24.7)	116 (26.6)
3	6025 (21.1)	4197 (21.0)	1398 (21.9)	341 (20.1)	89 (20.4)
4	5453 (19.1)	3806 (19.1)	1215 (19.0)	348 (20.6)	84 (19.3)
5 (most deprived)	4995 (17.5)	3659 (18.3)	1047 (16.4)	237 (14.0)	52 (11.9)

a *All other data presented as* n *(%).*

b *Missing data,* n *= 747. CKD = chronic kidney disease. eGFR = estimated glomerular filtration rate. SD = standard deviation. SIMD = Scottish Index of Multiple Deprivation.*

There were 670 drugs with a renal impairment warning in the BNF, of which 224 (33.8%) were examined and 446 excluded, with the most common reason being that the warning was too non-specific to measure (for example ‘use caution’ or ‘adjust dose’ without further specification) (Figure 1, Supplementary Tables S1–S6). Of the drugs for which a specific recommendation was included in the analysis, ‘avoid’ recommendations were made for 148 (22.1%) contraindicated drugs, ‘avoid if possible’ recommendations for 42 (6.3%) PHR drugs, and dose reduction recommendations for 34 (5.1%) DI drugs. The majority of contraindicate advice was specific to CKD stages 4 and 5.

**Figure 2.**
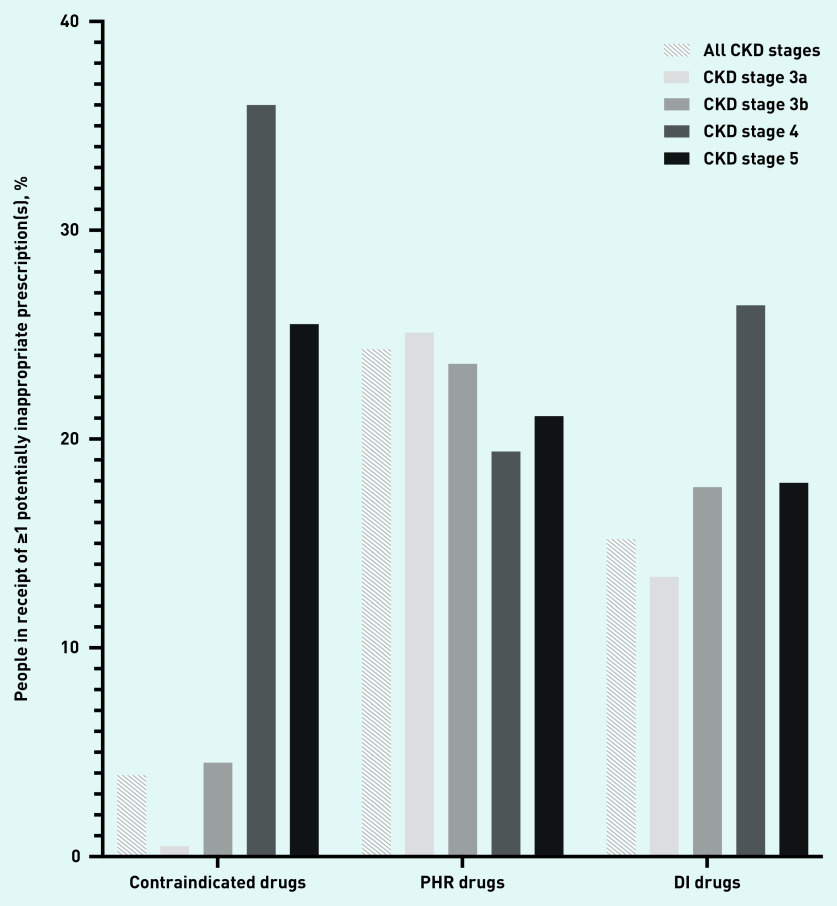
*Prevalence of potentially inappropriate prescribing by drug group and CKD stage.* *CKD = chronic kidney disease. DI = dose known to be inappropriate. PHR = potentially high risk.*

A total of 3.9% (95% CI = 3.7 to 4.1) of people with CKD stages 3a–5 were prescribed *≥*1 contraindicated drug, 24.3% (95% CI = 23.8 to 24.8) a PHR drug, and 15.2% (95% CI = 14.8 to 15.6) a DI drug (Table 2). Contraindicated drugs were least commonly prescribed throughout all CKD stages and were least common in CKD stage 3a, associated with fewer contraindicate restrictions being placed on drug use in this stage of CKD (Figure 2). In absolute terms, PHR drug prescriptions were most common in all stages of CKD, followed by DI drug prescriptions, with prescription of PHR drugs most common in CKD stage 3a and DI drugs in CKD stage 4.

**Table 2.** Prevalence of potentially inappropriate prescribing by CKD stage

**Drug group**	**Percentage of people in receipt of a prescription, % (95% CI)**				

	**All CKD stages, *n*= 28 489**	**CKD stage 3a, *n*= 19 977**	**CKD stage 3b, *n*= 6383**	**CKD stage 4, *n*= 1693**	**CKD stage 5, *n*= 436**
**Contraindicated drugs**					
≥1 drug	3.9 (3.7 to 4.1)	0.5 (0.4 to 0.6)	4.5 (4.0 to 5.0)	36.0 (33.7 to 38.2)	25.5 (21.5 to 29.5)
Most common drugs		Oxytetracycline 0.2 (0.1 to 0.3)	Nitrofurantoin 3.7 (3.2 to 4.2)	Aspirin 19.1 (17.2 to 21.0)	Aspirin 13.1 (9.9 to 16.2)
		Acetazolamide 0.06 (0.02 to 0.10)	Leflunomide 2.3 (1.1 to 3.6)	Thiazide 5.7 (4.6 to 6.9)	Lercanidipine 2.3 (0.9 to 3.7)
		Calcitriol 0.05 (0.02 to 0.08)	Oxytetracycline 1.1 (0.3 to 1.9)	Spironolactone 4.4 (3.4 to 5.4)	Metformin 1.8 (0.6 to 3.1)
				Ropinirole 1.8 (0.0 to 3.1)	

**PHR drugs**					
≥1 drug	24.3 (23.8 to 24.8)	25.1 (24.5 to 25.7)	23.6 (22.5 to 24.6)	19.4 (17.6 to 21.3)	21.1 (17.3 to 24.9)
Most common drugs		Co-codamol 11.3 (10.9 to 11.8)	Co-codamol 9.6 (8.8 to 10.4)	Co-codamol 6.9 (5.6 to 8.2)	Oxycodone 6.2 (4.5 to 7.9)
		Tramadol 6.2 (5.9 to 6.6)	Tramadol 6.2 (5.6 to 6.8)	Oxycodone 6.2 (4.5 to 7.9)	Morphine 6.0 (3.2 to 8.7)
		Naproxen 3.5 (3.2 to 3.8)	Oxycodone 4.8 (4.0 to 5.6)	Tramadol 5.3 (4.2 to 6.4)	Co-codamol 5.3 (3.1 to 7.5)

**DI drugs**					
≥1 drug	15.2 (14.8 to 15.6)	13.4 (12.9 to 13.8)	17.7 (16.4 to 18.3)	26.4 (24.3 to 28.6)	17.9 (14.4 to 21.8)
Most common drugs		Ramipril 8.3 (7.9 to 8.6)	Ramipril 7.9 (7.2 to 8.6)	Simvastatin 10.0 (8.5 to 11.4)	Ranitidine 6.6 (4.3 to 9.0)
		Atorvastatin 2.8 (2.6 to 3.1)	Ranitidine 4.4 (3.9 to 4.9)	Ranitidine 5.1 (4.0 to 6.1)	Simvastatin 6.4 (4.1 to 8.7)
		Sitagliptin 1.5 (1.4 to 1.7)	Atorvastatin 2.9 (2.5 to 3.3)	Ramipril 4.3 (3.3 to 5.3)	Ramipril 4.3 (3.3 to 5.3)

*CI = confidence interval. CKD = chronic kidney disease. DI = dose known to be inappropriate. PHR = potentially high risk.*

### Prevalence of contraindicate prescribing by CKD stage

**Figure 3.**
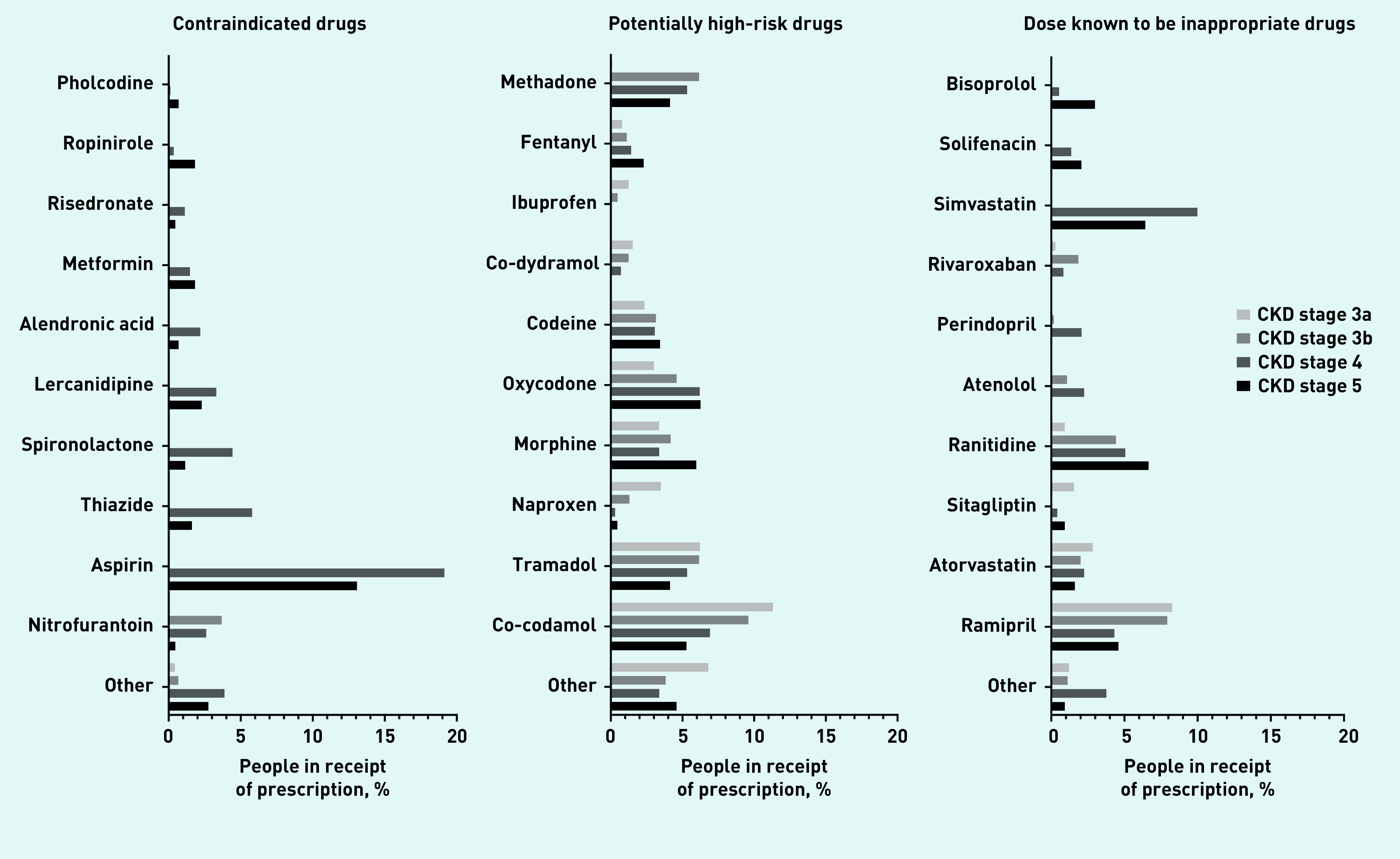
*Prevalence of potentially inappropriate prescribing by drugs within drug group.*

Prevalence rates for contraindicate prescribing differed substantially depending on CKD stage. Prescribing rates in all CKD stages were low (3.9%, 95% CI = 3.7 to 4.1). The lowest prevalence was in CKD stage 3a (0.5%, 95% CI = 0.4 to 0.6), and the most common prescription in this group was oxytetracycline (0.2%, 95% CI = 0.1 to 0.3) (Table 2, Figure 3, Supplementary Table S7). Prescribing rates rose to 4.5% (95% CI = 4.0 to 5.0) in CKD stage 3b, with nitrofurantoin prescribing accounting for 3.7% (95% CI = 3.2 to 4.2) of this figure. The majority of BNF contraindicate recommendations related to CKD stage 4 or worse (Figure 1), and people with CKD stage 4 had the highest prevalence of contraindicate prescribing (36.0%, 95% CI = 33.7 to 38.2). The most commonly prescribed contraindicated drug in CKD stages 4 and 5 was aspirin (19.1%, 95% CI = 17.2 to 21.0, and 13.1%, 95% CI = 9.9 to 16.2, respectively) (Table 2, Figure 3, Supplementary Table S7). Prescribing rates were similar between sexes, most common in the age group *≥*85 years, and similar throughout all SIMD quintiles (Table 3).

**Table 3.** Prevalence of potentially inappropriate prescribing by sex, age, and socioeconomic status

	***n***	**People in receipt of a prescription, % (95% CI)**		

		**Contraindicated drugs**	**PHR drugs**	**DI drugs**
**Sex**				
Female	17 768	4.4 (3.0 to 5.8)	5.7 (4.3 to 7.1)	14.3 (13.0 to 15.5)
Male	10 721	4.2 (2.4 to 6.0)	4.6 (2.8 to 6.4)	22.1 (20.7 to 23.6)

**Age, years**				
18–24	21	0.0	0.0	0.0
25–34	152	3.3 (0.0 to 18.7)	12.5 (0.0 to 26.5)	7.2 (0.0 to 22.0)
35–44	369	2.4 (0.0 to 12.4)	8.4 (0.0 to 17.8)	11.9 (5.8 to 18.1)
45–54	1367	2.8 (0.0 to 8.0)	9.4 (4.6 to 14.2)	15.4 (10.9 to 19.9)
55–64	3285	2.5 (0.0 to 5.9)	9.8 (6.8 to 12.4)	17.3 (14.4 to 20.1)
65–74	7509	3.6 (1.4 to 5.8)	7.0 (4.9 to 9.1)	20.3 (18.6 to 22.2)
75–84	9478	4.4 (2.5 to 6.3)	3.8 (1.9 to 5.7)	19.3 (17.7 to 20.9)
≥85	6308	6.4 (4.1 to 8.7)	1.9 (0.0 to 4.4)	11.4 (9.2 to 13.6)

**Socioeconomic status by SIMD quintile^a^**				
1 (least deprived)	4981	4.6 (1.9 to 7.2)	5.4 (2.8 to 8.1)	18.9 (16.7 to 21.1)
2	6288	4.3 (1.9 to 6.7)	5.1 (2.8 to 7.4)	18.3 (16.3 to 20.3)
3	6025	4.5 (2.1 to 6.9)	5.9 (3.5 to 8.3)	17.1 (15.0 to 19.2)
4	5453	4.8 (2.3 to 7.3)	4.9 (2.4 to 7.4)	16.3 (14.1 to 18.6)
5 (most deprived)	4995	3.2 (0.5 to 5.8)	5.0 (2.4 to 7.7)	15.7 (13.4 to 10.1)

a *Missing data,* n *= 747. CI = confidence interval. DI = dose known to be inappropriate. PHR = potentially high risk. SIMD = Scottish Index of Multiple Deprivation.*

### Prevalence of PHR drugs

All stages of CKD had similar prevalence rates for PHR prescribing. Lowest prevalence was seen in CKD stage 4 (19.4%, 95% CI = 17.6 to 21.3), and highest in CKD stage 3a (25.1%, 95% CI = 24.5 to 25.7) (Table 2, Figure 3). Co-codamol was the most commonly prescribed PHR drug in CKD stages 3a (11.3%, 95% CI = 10.9 to 11.8), 3b (9.6%, 95% CI = 8.8 to 10.4), and 4 (6.9%, 95% CI = 5.6 to 8.2). Oxycodone was the most frequently prescribed PHR drug in CKD stage 5 (6.2%, 95% CI = 4.5 to 7.9). The most commonly prescribed non-steroidal anti-inflammatory drug (NSAID) was naproxen, with prescribing prevalence of 3.5% (95% CI = 3.2 to 3.8) in CKD stage 3a, 1.3% (95% CI = 1.0 to 1.6) in CKD stage 3b, 0.3% (95% CI = 0.04 to 0.6) in CKD stage 4, and 0.5% (95% CI = −0.2 to 1.1) in CKD stage 5 (Table 2, Figure 3, Supplementary Table S8). Prescribing rates for NSAIDs such as naproxen, ibuprofen, and diclofenac decreased as CKD stage increased (see Supplementary Table S8). Prescribing rates were similar between sexes, were most common in the 45–64 years age group, and were similar throughout all SIMD quintiles (Table 3).

### Prevalence of DI drugs

Excessive dosing was least common in CKD stage 3a at 13.4% (95% CI = 12.9 to 13.8), and most common in CKD stage 4 (26.4%, 95% CI = 24.3 to 28.6) (Table 2, Figure 3, Supplementary Table S9). Ramipril was the most commonly prescribed DI drug in CKD stage 3a (8.3%, 95% CI = 7.9 to 8.6) and 3b (7.9%, 95% CI = 7.2 to 8.6). Simvastatin was the most frequently prescribed DI drug in CKD stage 4 (10.0%, 95% CI = 8.5 to 11.4), and was not seen in earlier stages of CKD due to dose instructions being specific to CKD stage 4 and worse. Ranitidine was the most commonly prescribed DI drug in CKD stage 5 (6.6%, 95% CI = 4.3 to 9.0) (Table 2, Figure 3). Prescribing rates were significantly higher in males than females, most common in the 65–74 years age group, and similar throughout all SIMD quintiles (Table 3).

## DISCUSSION

### Summary

In this large primary care-based study, potentially inappropriate prescribing was widespread at all stages of CKD. Contraindicated drugs represented the least common potentially inappropriate drug prescribing to people with all stages of CKD, and there was substantial variation in prescribing rates by CKD stage, with most of this prescribing being seen in CKD stages 4 and 5. PHR drugs were the most commonly prescribed drugs throughout all stages of CKD, showing much less variation between CKD stages. DI drugs were commonly seen in all stages of CKD, showing highest prescribing prevalence in CKD stage 4.

### Strengths and limitations

Strengths of this study include the systematic analysis of potentially inappropriate prescribing in primary care for people with known CKD within a population cohort, with ascertainment of CKD using laboratory data and measurement of dispensed prescribing.

Limitations include the absence of clinical details such as comorbidities, and physical parameters such as blood pressure readings and urinalysis findings, which would have allowed better evaluation of the appropriateness of prescribing and address the difficult decisions faced by GPs when weighing up the risks and benefits of prescribing. Inclusion of prescribing site and individual physician prescribing practices would have provided relevant information to support the development of interventions to improve prescribing safety; however, data for these areas were not available within the dataset. CKD stage was defined by eGFR rather than directly measured, but this is inevitable in a large clinical dataset, and GFR was estimated using standardised creatinine for consistency. The study only examined prescribed drugs, and patients can purchase some nephrotoxic drugs from pharmacists (notably NSAIDs). Calculating the dose of a drug taken using routine data is difficult. For the DI drugs, the authors therefore only report prescribing when they can be certain that the dose was inappropriate based on the strength dispensed. In addition, the prevalence of CKD is based on the laboratory information available to the clinician, which means some people within the population will remain undiagnosed. Therefore, prescribing rates in this study are conservative and the prevalence of potentially inappropriate prescribing will be worse than reported. However, very high proportions of older people had at least two eGFR values, so the authors do not expect under-ascertainment to be too serious, given that CKD prevalence is most common in this group. Finally, renal impairment warnings in the BNF are frequently nonspecific, meaning that the authors could not reliably measure the appropriateness of prescribing for the majority of the drugs with any renal warning, reflecting the ambiguity in the evidence. However, the finding that clinicians are commonly expected to use clinical judgement in the face of minimal evidence is an important one in its own right.

### Comparison with existing literature

Several studies refer to potentially inappropriate prescribing in secondary care,^25^^–^^27^ but few studies examine primary care prescribing. A recent primary care-based study by Wood *et al* reported prescribing outside recommendations of 2.0%–39.9% in a sample of eight drugs.^18^ Angiotensin-converting enzyme inhibitors (ACEi), simvastatin, thiazides, NSAIDs, and metformin were commonly prescribed, drugs that were also commonly seen in the current study population. Byrne *et al* examined nine high-risk prescribing combinations, demonstrating significant variation in potentially inappropriate prescribing practice between individual GP prescribers, and found that 15% of patients vulnerable to adverse drug events (ADEs) received *≥*1 high-risk prescriptions over a 1-year period.^19^ A Swedish large population primary care study analysed renally inappropriate prescribing in older people with renal impairment.^21^ It identified similar patterns of potentially inappropriate prescribing to that found in the current study, including ACEi, simvastatin, metformin, opioids, and NSAIDs. One serum creatinine measurement was used to identify the CKD cohort and the study found a prevalence of inappropriate prescribing of 42.5% and 58.1% for CKD stages 3 and 4, respectively. The higher prevalence likely reflects the use of a 1-year look-back period for prescribing compared to 84 days in the current study. A North American primary care study looked at the number and proportion of adults with CKD stages 3 and 4 who were prescribed *≥*1 NSAID or another relatively contraindicated medication.^20^ It examined prescribing over a 2-year period and found that 46.6% were prescribed a relatively contraindicated drug, and 34.0% an NSAID during the study period. Hull *et al* performed a cross-sectional survey of 12 011 patients with CKD in a population in England, examining NSAID prescribing rates by ethnicity, and found that prescribing rates decreased with increasing CKD stage in people of all ethnicities,^28^ a finding that was also noted in the present study. Prescribing of specific drugs has been seen in the current study and across other similar observational studies, indicating the strength of this evidence base and the applicability of this study’s findings to clinical decision making and health policy. Study design among existing literature is highly heterogeneous, making it difficult to make direct comparisons and identify clear conclusions; however, it is clear that potentially inappropriate prescribing in the primary care setting is a significant problem.

### Implications for research and practice

Many drugs were prescribed outside BNF renal prescribing recommendations, but some of this prescribing is recommended in other clinical guidance. Notably, the BNF recommends avoiding aspirin in severe renal impairment (for the purposes of this study interpreted as CKD stages 4 and 5), and this was the single most commonly prescribed potentially inappropriate drug in the study. However, given high rates of CVD in people with CKD, the indication for aspirin is usually very strong. A balance of benefit and risk that depends on strength of indication as well as evidence of risk for each individual is needed, and clinicians face difficult decisions to weigh up the risk-to-benefit ratio in individuals.^29^ High-risk prescribing can be appropriate if the benefits outweigh the potential harm of omitting a drug,^30^ such that the correct indicator for these prescribing rates is unlikely to be zero. Additional pharmacoepidemiology studies in the context of CKD are needed to provide a stronger evidence base.

Research is needed to better understand processes associated with prescribing and improve existing mechanisms for making prescribing safer, including acute and repeat prescribing practices, and exploring analgesic use in palliative care. Evaluation of prescribing practices between GP practices would also provide useful information on which to base a complex intervention. A UK primary care-based study showed that a combination of professional education, clinician prompts, and financial incentives significantly reduced the rate of high-risk prescribing of NSAIDs and antiplatelet medications, supporting use of complex interventions to reduce high-risk prescribing.^30^ At present, Scottish GP electronic medical records prescribing systems do not trigger point-of-care alerts to clinicians based on the presence of renal impairment. Alerts based on renal function might improve prescribing safety, and this is an important area for evaluation in future research. Decisions to stop medications can be patient dependent, with some patients preferring to accept the risks of harm from certain medicines, particularly those that improved quality of life, in the context of informed discussions where patients are exerting choice over treatment. Increasing the time available for GPs and pharmacists to engage with medication reviews might be related to improving the use of medications, for example, reducing potentially inappropriate prescribing without a clear indication.^31^

This study has provided a systematic examination of potentially inappropriate prescribing in known CKD in the primary care setting. Existing recommendations for prescribing in renal impairment are often non-specific and relatively unhelpful to clinicians. There is a need for research to improve understanding of the benefit–harm balance of prescribing in renal impairment, and to develop and evaluate interventions to improve prescribing safety in this population.
